# Normothermic Regional Perfusion is an Emerging Cost-Effective Alternative in Donation After Circulatory Death (DCD) in Heart Transplantation

**DOI:** 10.7759/cureus.26437

**Published:** 2022-06-29

**Authors:** Emad Alamouti-fard, Pankaj Garg, Ishaq J Wadiwala, John H Yazji, Mohammad Alomari, Md Walid Akram Hussain, Mohamed S Elawady, Samuel Jacob

**Affiliations:** 1 Cardiothoracic Surgery, Mayo Clinic, Jacksonville, USA; 2 Colorectal Surgery, Mayo Clinic, Jacksonville, USA

**Keywords:** cold storage, warm ischemia, donors pool, heart transplantation, normothermic regional perfusion (nrp), donation after circulatory death (dcd)

## Abstract

In donation after circulatory death (DCD) organ transplantation, normothermic regional perfusion (NRP) restores oxygenated blood flow following cardiac arrest and reverses warm ischemia. Recently, NRP has also been used to help recover DCD hearts in addition to the abdominal organs. While DCD donation has increased the number of abdominal organs and lungs pool, it has not been able to increase the number of heart transplants, despite the fact that it has the potential to increase the number of heart transplants by 15-30%. Thoracoabdominal normothermic regional perfusion makes heart transplantation feasible and permits assessing heart function before an organ procurement without affecting the preservation of abdominal organs. NRP can be used in two ways for DCD donor heart transplants: normothermic regional perfusion followed by machine perfusion (NRP-MP) and normothermic regional perfusion followed by static cold storage (NRP-SCS). Normothermic regional perfusion is an emerging technology, a cost-effective alternative in donation after circulatory death (DCD), and will increase the pool of donors in heart transplantation.

## Introduction and background

Heart transplant remains the gold standard for the treatment of heart failure; however, there is a shortage of donors' hearts compared to the ever-increasing waitlist of recipients. Presently, donation after brain death (DBD) remains the most common method of organ donation [1,2]. Nevertheless, a renewed interest has emerged in recent decades in donating organs from patients who are not brain dead as per the criteria but whose neurological injuries are severe enough for them not to have a meaningful, productive life. Death in such donors is established by circulatory and respiratory criteria instead of brain death after withdrawing the life-sustaining support in a controlled environment, and donation is done after circulatory death (DCD).

The concept of heart transplants after DCD donation is not new. The first heart transplant performed by Christian Bernard in 1967 was also used as a DCD heart. After the establishment of brain death criteria in 1968, donation after brain death (DBD) donors remained the only source of heart donation for nearly 36 years [3]. However, with an increase in the prevalence of heart failure and an increase in the number of patients on the waitlist for a heart transplant, the search for newer methods to increase the donor pool has started in the last two decades. In 2018, about 20% of organ donations in the United States came from DCD donors [4,5]. The concept of controlled DCD (cDCD) donation refers to the donations made by deceased individuals after the withdrawal of life-sustaining therapies because it is no longer deemed to be in their best interests [6]. In DCD donation, life-sustaining support, especially ventilation, is withdrawn to allow the donor to have circulatory arrest spontaneously and organs are procured after five minutes of electromechanical quiescence. Generally, DCD results in fewer organs per donor, and the quality of organs is less compared to donation after brain death (DBD) due to the period of warm ischemia surrounding the arrest [7]. There are certain concerns with DCD donations. One of the concerns about the graft functionality of the DCD heart is that the heart undergoes a period of warm ischemia during the withdrawal of life-sustaining therapy (WLST) [8]. So far, studies have been unable to predict the time point at which myocardial injury is established, which results in graft dysfunction and when the myocardial cell damage becomes irreversible [9]. According to recent studies, WLST seems to maintain myocardial contractility and cellular viability for the first 10 minutes after cardiac arrest and beyond 10 minutes, heart graft function is likely to be compromised [8].

Donation after DCD has increased the pool of abdominal organs and lungs, but its impact on the heart donor pool is still meager, despite the fact that it can increase the number of heart transplants by 15-30% and reduce the death rate in the hospital's waiting list by 40% [10]. Noterdaeme et al. demonstrated in their study that DCD hearts that met criteria (DBD criteria + donation withdrawal ischemia time less than 30 minutes) could increase the number of heart transplants by 11% [10]. According to another study, DCD hearts could increase the heart transplant pool by 30% [11]. These studies show that DCD donation can significantly increase the heart donation pool. In this review, we will discuss NRP, its conduct, potential applications, and shortcomings. In the end, we will discuss the future directions.

## Review

Organ recovery and protection

For the DCD donation, as per our institutional protocol, we wait for the maximum duration of 27 minutes from the time of systolic arterial pressure when it is below 50 mmHg to electromechanical arrest. If the donor does not have an electromechanical arrest within this time, we decline the heart. If the electromechanical arrest happens within 27 minutes, after five minutes of wait time, the chest is entered swiftly. After widely spreading the sternum with a retractor, the pericardium is opened in less than a minute. Then, there are two methods to recover the heart. In the first method, the right atrium (RA) is cannulated with a large two-stage venous cannula, and 1100cc to 1500cc of blood is collected in blood collecting bags in less than 1 to 1.5 minutes followed by aortic cross-clamping and delivery of cold cardioplegia into the aortic root. In our institute, we prefer to use Celsior crystalloid cardioplegia (Genzyme Corp., Boston, MA). After the delivery of cardioplegia, the heart recovers. In the second method, the aorta and right atrium are cannulated, and modified veno-arterial extracorporeal membrane oxygenation (VA-ECMO) is instituted called normothermic regional perfusion (NRP). In either method, the duration from the skin incision to the initiation of cardioplegia or NRP should be less than three minutes.

In cases where the heart is immediately recovered after infusing the cold cardioplegia into the aortic root, the recovered heart can be transported by two methods. (1) Transport the heart after direct cold preservation (DCP). This technique is rapid. However, as heart function is never visualized after the cardiac arrest, there is always concern about the recovery of heart function after the transplantation. This method is seldom used nowadays. Still, if the donor and recipient are in the same facility and a resource-limited setup, this technique may be useful. (2) Connect the recovered heart to the organ care system (OCS) after the procurement to allow coronary perfusion. This allows the heart to recover from the warm ischemic injury and also allows the real-time assessment of the heart function. The main drawbacks of the OCS heart machine are: (1) the OCS machine is large and needs a large space for transport, so it becomes a challenge, especially in air transport. (2) It needs a lot of trained personnel to establish the OCS machine and is also cost-intensive.

Normothermic regional perfusion

**Figure 1:**
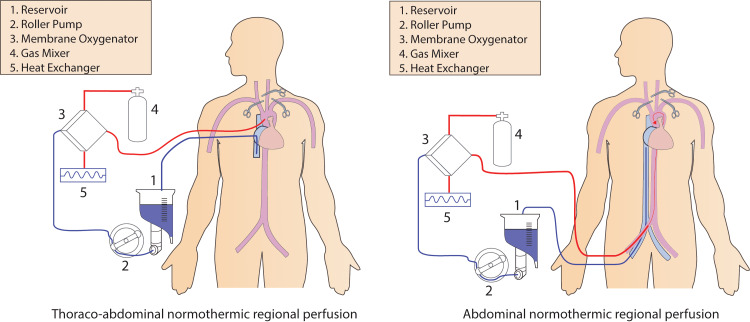
Cannulation and circuit for normothermic regional perfusion. VA-ECMO circuit is the same for both TA-NRP and A-NRP. In TA-NRP, the aorta and right atrium are cannulated; in A-NRP, the femoral artery and femoral vein are cannulated. In addition, the circuit is modified to accommodate two suctions for blood collection. VA-ECMO: veno-arterial extracorporeal membrane oxygenation, TA-NRP: thoracoabdominal normothermic regional perfusion, A-NRP: abdominal-normothermic regional perfusion.

Normothermic regional perfusion is a method of maintaining the allografts after the DCD donation by instituting the VA-ECMO to maintain the thoracic and organ perfusion to allow them to have time to recover from the warm ischemic injury. Normothermic regional perfusion is of two types depending upon the organs planned to be recovered. In donors where the heart, as well as other abdominal organs, are planned to be recovered, thoracoabdominal normothermic regional perfusion (TA-NRP) is performed, while in donors with only abdominal organ recovery, abdominal-NRP (A-NRP) is performed [3,12,13]. The different cannulation and circuit methods are illustrated in Figure 1.

Circuit and conduct of normothermic regional perfusion

**Figure 2:**
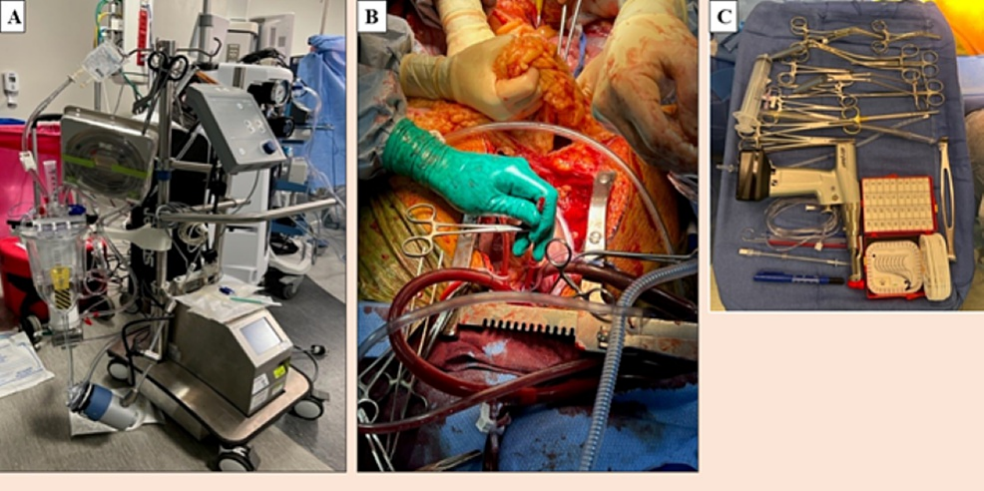
Equipment used in normothermic regional perfusion. Showing the circuit (A) and cannulation (B) of thoracoabdominal normothermic regional perfusion. (C) shows the required instruments setup on the Mayo stand (USA Medical and Surgical Supplies, St. Louis, MO).

In both TA-NRP and A-NRP, the circuit is the same except that in TA-NRP, central cannulation of the aorta and right atrium is done, while in A-NRP, femoral artery and vein or abdominal aorta and inferior vena cava (IVC) near bifurcation are cannulated. In both TA-NRP and A-NRP, the circuit consists of a VA-ECMO circuit, i.e., venous drainage cannula size 29 fr, arterial flow cannula size 19 fr connecting tubing, and a centrifugal pump (Figures 2A, 2B). However, like the cardiopulmonary bypass (CPB) circuit, it contains the reservoir to drain the blood and keep the heart empty. To prime the NRP circuit, four units of washed and packed red blood cells should be carried along with the circuit from the recipient hospital. Prior to the withdrawal of life support for a DCD donor, the Mayo table should be setup with all the required instruments (Figure 2C). In both the NRP systems, 30,000 units of heparin are given to the donor before the withdrawal of support.

In A-NRP, blood is drained from the venous circuit from the IVC, oxygenated, and returned to the abdominal aorta. Also, a Coda balloon catheter (Cook Incorporated, Bloomington, IN) is inserted through the aortotomy site and inflated in the thoracic aorta or abdominal aorta is clamped just below the diaphragm to prevent blood from escaping into the thoracic organs and brain. The suprahepatic inferior vena cava is clamped to prevent the recirculation of thoracic and cerebral blood. In our institute, we continue the perfusion of the abdominal organs for 60 minutes. At the end of the 60 minutes, if the organs are suitable for transplant, NRP perfusion is stopped, the organs are perfused with the University of Wisconsin (UW) solution, and organs are recovered [12].

In TA-NRP, the venous cannula is inserted into the RA and the arterial cannula is inserted into the ascending aorta after clamping the innominate and left common carotid arteries. The NRP circuit for TA-NRP is similar to the A-NRP circuit (Figures 2C, 2D). Usually, the heart starts beating two to three minutes after initiating the perfusion. The heart is visually inspected and supported with inotropes or vasopressors or dilators if required to maintain the mean perfusion pressure between 60 mmHg and 80 mmHg. As the cardiac contractility improves, all pressors and inotropes are weaned. Serum lactate is checked to confirm adequate organ perfusion and electrolytes are checked to rule out hypokalemia or hyperkalemia. After perfusion for 30 to 60 minutes depending upon the cardiac contractility and weaning off all inotropes and vasopressors, NRP is gradually weaned continuously assessing the myocardial contractility. After completely weaning the NRP, if cardiac contractility is adequate and the decision is made to procure the heart, the venous cannula is removed. We prefer to leave the aortic cannula in place as removing the aortic cannula significantly increases the risk of aortic cannulation bleeding in a heparinized patient. After that, the heart and abdominal organs are mobilized as in DBD donation. After communication between the thoracic and abdominal teams, the aorta is cross clamped, aortic root cardioplegic delivered, and the heart is recovered [3]. Figure 2 shows the different equipment used for NRP. In TA-NRP, the abdominal organ procurement team does not need to hurry to open the abdomen as after establishing the NRP, perfusion is established to both the thoracic and abdominal organs. Further, it decreases the risk of ischemic cholangiopathy.

Conduct of perfusion

For both TA-NRP and A-NRP, after the quiescence of electromechanical activity and five minutes of wait time, the chest and abdomen (TA-NRP) or abdomen (A-NRP) are entered quickly. In TA-NRP, sternal edges are separated with a retractor, the pericardium is opened, the aorta and right atrium cannulated, the innominate artery and left common carotid artery clamped, and VA-ECMO flow established. In A-NRP, the abdominal aorta and inferior vena cava are cannulated and VA-ECMO is established. The Coda balloon catheter is passed through the same aortotomy and inflated in the descending thoracic aorta or abdominal aorta at the diaphragm and suprahepatic inferior vena are clamped to prevent the circulation of blood into the thoracic organs and brain [13]. We aim to keep the duration from skin incision to the initiation of NRP to less than two to three minutes. 

Advantages of normothermic regional perfusion

The advantages of NRP are: (1) as a result of continuous warm blood perfusion, NRP restores heart function, reduces myocardial injury, promotes energy storage, and maintains homeostasis; (2) visual assessment of the heart and abdominal organs; (3) allowing the heart and other organs to recover by establishing organ perfusion; (4) it also reduces the time spent by the organ in warm ischemia; and (5) it enables the assessment of the viability of the heart and other organs in a non-ischemic state (compared to direct cold storage) before retrieval [13,14].

The advantage of TA-NRP over A-NRP is that once spontaneous sinus rhythm returns and NRP is weaned off, the abdominal organs are perfused with pulsatile and physiological perfusion pressure through the heart function rather than being perfused with continuous pressure and blood flow through abdominal NRP. There is a possibility that this physiological abdominal recirculation could minimize the need for additional multiple and costly ex-situ perfusion devices for each individual organ [15]. Studies have shown that NRP prior to recovery for DCD liver donation reduces the risk of cholangiopathy in the donor's liver [13] and earlier recovery in renal function after transplantation compared to in-situ cold perfusion [16]. As a consequence, the majority of transplant centers are moving away from rapid cold preservation and recovery following death (the "gold standard" for DCD organ recovery) in favor of normothermic regional perfusion (NRP) to temporarily restore oxygenated blood flow to abdominal and, more recently, thoracic organs before recovery [12,13].

Post-recovery heart management

For the DCD heart procurement, after NRP and recovery of the heart after arresting with cold crystalloid cardioplegia, myocardial protection can be continued by two techniques. First, normothermic regional perfusion followed by machine perfusion (NRP-MP) [17], and second, normothermic regional perfusion followed by static cold storage (NRP-SCS) [18,19]. Previously, DCD heart transplantation was limited by the requirement for the donor and the recipient to be in the same location. However, NRP with both these techniques allows us to overcome this limitation [20]. Further, the use of TA-NRP in DCD heart donors in conjunction with cold storage following retrieval can abate the need for ex-situ [15] OCS, making DCD heart transplantation economically viable even in resource-limited countries also.

However, using OCS after NRP has several advantages over NRP-SCS. These include: (1) the heart can be transported for long distances and OCS reduces the cold ischemia time significantly. While the use of ice has been employed for a long time for the preservation of organs, novel machines have recently been invented for better preservation. One of them is the Sherpa Pak (SherpaPak™, Paragonix Technologies, MA, USA), which got the "green light" to be utilized for organ preservation since 2018. This machine uses a single-use disposable sterile box where the heart can be preserved at a temperature ranging from 4°C to 8°C. This allows for more predictable and stable maintenance of temperature. Preliminary studies have shown improved graft survivability when organs were preserved and transported using Sherpa Pak instead of ice for cold static storage [21]. (2) In recipients requiring complicated mediastinal dissection, e.g., in patients with prior left ventricular assist device implantation, prior surgery for congenital heart defects, OCS provides sufficient time for a safe dissection and cardiectomy even after the donor's heart reaches the recipient hospital.

Direct procurement and perfusion (DPP) is another technique for DCD donors that can be compared to NRP. In DPP, the cold ischemia time begins as soon as we deliver the cold cardioplegia, after cross-clamping the aorta. This obviates the need for the collection of donor blood prior to the delivery of cardioplegia [22]. However, in contrast to the DBD patient or DCD hearts procured after NRP, in the DPP technique, no evaluation of cardiac function can be done prior to removing the cross-clamp after the heart transplantation in the recipient. Messer et al. compared the outcomes between DCD heart transplants performed with DPP and NRP, they showed no significant difference in outcomes with the two techniques [17,23]. However, due to a paucity of studies, this area needs to be investigated further. Else, after direct procurement, the heart can be perfused with an expensive ex-vivo OCS perfusion system to assess the heart function. In contrast, NRP permits rapid reperfusion and assessment of allograft function under physiological conditions. Smith and colleagues have demonstrated promising outcomes, including weaning all donor hearts off of CPB without inotropes, a 100% recipient survival rate with a median follow-up of approximately one year, a post-discharge left ventricular ejection fraction of 64%, and no patients requiring mechanical circulation support [24]. Although NRP is resource-intensive and will cost an additional $4000 for each heart assessed for equipment and personnel, compared to OCS, it is more feasible. The OCS cost includes console costs of about $275,000 USD and the single-use components cost about $38,000-$55,000 USD. In addition, the system requires a maintenance service that will cost about $20,000 AUD (2016 dollars) over a 10-year period. Studies demonstrate that 100% of hearts from NRP donors have been used once they have been assessed and accepted for transplantation, compared to 17% of DPP hearts that were turned down for transplantation after perfusion on the OCS at a potential cost of $114,000 each. The rate of turndown is consistent in several studies [17,25].

Limitations

The DCD heart transplantation with NRP needs to overcome several important obstacles before it can become a mainstream procedure. Resolving these concerns can have a remarkable effect on using DCD donors and expanding the heart transplant donor pool. The NRP, as described by the investigators, is logistically challenging and requires considerable coordination between the donor hospital, procurement teams, and perfusionists, as well as the organ procurement organization (OPO). Successful implementation of the protocol requires the agreement of all parties. Donor and recipient locations are concerns [26], but we can overcome them with machine perfusion or cold storage to relocate the allografts.

Ethical issues

Both DCD and DBD donor procurement procedures were and always be an ethical dilemma. Some countries, such as Australia, prohibit this practice. Existing laws of the USA support organ recovery professionals engaged in the lifesaving work of DCD that occurs today, including NRP [27]. As the debate around the ethical aspects of NRP heart procurement continues, it is of paramount importance that clear communication takes place with donor families to avoid misunderstandings, as the trust of the donors and their families is at risk [26].

It has been well established for the last six decades that brain death is ethically equivalent to biological death, as the brain is essential to performing the important work of integrating the functions of the body. Without it, the person as an organism would not be able to function at all. Circulatory death requires "an irreversible cessation of circulatory and respiratory functions." Some critics of cDCD have asserted that such donors are alive at the time of organ recovery since circulation can be re-established after five minutes of circulatory arrest, so it is not irreversible. In a counterargument, it is argued that if there are no plans to restore circulation, the patient is dead (even if potentially reversible) [28,29]. Death according to neurological criteria and death according to circulatory criteria share a thread of loss of all brain function, according to the "unifying concept of death" [30,31]. As a result of circulatory death, the loss of circulation represents the permanent destruction of the brain, and the devastation of the brain is irreversible [28].

## Conclusions

DCD heart transplantation can significantly increase the pool of heart donors and make it possible to have more hearts available for a heart transplant. However, there are not many studies that compared NRP to DPP and ex-vivo reperfusion. Comparison, mortality rates, and complications must be investigated in further studies to have a better understanding of the benefits and disadvantages of these techniques.
